# Lysosome Transport as a Function of Lysosome Diameter

**DOI:** 10.1371/journal.pone.0086847

**Published:** 2014-01-31

**Authors:** Debjyoti Bandyopadhyay, Austin Cyphersmith, Jairo A. Zapata, Y. Joseph Kim, Christine K. Payne

**Affiliations:** School of Chemistry and Biochemistry and Petit Institute for Bioengineering and Bioscience, Georgia Institute of Technology, Atlanta, Georgia, United States of America; National Institute of Biological Sciences, Beijing, China

## Abstract

Lysosomes are membrane-bound organelles responsible for the transport and degradation of intracellular and extracellular cargo. The intracellular motion of lysosomes is both diffusive and active, mediated by motor proteins moving lysosomes along microtubules. We sought to determine how lysosome diameter influences lysosome transport. We used osmotic swelling to double the diameter of lysosomes, creating a population of enlarged lysosomes. This allowed us to directly examine the intracellular transport of the same organelle as a function of diameter. Lysosome transport was measured using live cell fluorescence microscopy and single particle tracking. We find, as expected, the diffusive component of intracellular transport is decreased proportional to the increased lysosome diameter. Active transport of the enlarged lysosomes is not affected by the increased lysosome diameter.

## Introduction

Lysosomes are membrane-bound organelles essential for endocytosis, phagocytosis, and autophagy [1]–[3]. Fusion of endosomes, phagosomes, and autophagosomes with lysosomes exposes the cargo in these organelles to the hydrolytic enzymes and low pH of the lysosomes resulting in the degradation of cargo. Like many organelles, the diameters of lysosomes are heterogeneous. The canonical lysosome diameter ranges from 50 nm to 500 nm [1]. A fundamental question is how the size of the lysosome affects intracellular transport.

Lysosome mobility is a combination of active transport and diffusion [3]–[6]. Active, ATP-dependent, transport is driven by motor proteins, kinesin and dynein, moving the lysosome along microtubules. Lysosomes also undergo periods of diffusion. Diffusion can be free or constrained by cellular structures, leading to sub-diffusive behavior. Lysosome diffusion should scale inversely with the diameter of lysosomes, with the caveat that viscosity can vary as a function of sub-cellular environment or cell cycle [7], [8]. It is less clear how lysosome diameter affects active transport. It is possible that the viscosity of the cytosol results in a diameter-dependent drag force on the lysosome. Previous results have shown that organelle transport varies as a function of number of motor proteins for peroxisomes in insect cells [9], vesicles in model neurons [10], and melanosomes in Xenopus cells [11]. These results suggest that cytosolic drag is an important factor in intracellular transport. However, conflicting results have been obtained showing the kinesin-mediated transport of lipid droplets in Drosophila embryos is independent of the number of motors [12]. Overall, the importance of cytosolic drag on intracellular transport has been controversial [13]–[15].

Our goal was to determine how lysosome diameter affects lysosome transport within live cells. In order to measure the transport of lysosomes as a function of diameter, we increased the diameter of lysosomes by incubating cells with sucrose. Sucrose accumulates in lysosomes, but is not degraded within the lysosomes, resulting in an osmotic swelling of the lysosome [16]–[18]. Sucrose incubation resulted in a population of enlarged lysosomes with diameters above the diffraction limit that allowed us to measure lysosomal transport for small, untreated, lysosomes and enlarged, sucrose-swollen, lysosomes. Normal and enlarged fluorescently-labeled lysosomes were tracked during intracellular transport using live cell fluorescence microscopy. Single particle tracking shows that, as expected, diffusion decreased for larger lysosomes. Active transport, both anterograde and retrograde, was not affected by the increased lysosome diameter.

Understanding the intracellular transport of lysosomes is necessary to understand cellular function as well as the human diseases associated with lysosomal disruption or defects [2], [19], [20]. In addition, this research addresses the broader question of how organelle size affects transport. Our results for lysosomes can be easily extended to other transport vesicles including early and late endosomes, clathrin-coated vesicles, and caveosomes as well as the viruses, nanoparticles, nutrients, and cell surface receptors that are transported within these vesicles.

## Results

### Increasing Lysosome Diameter

**Figure 1:**
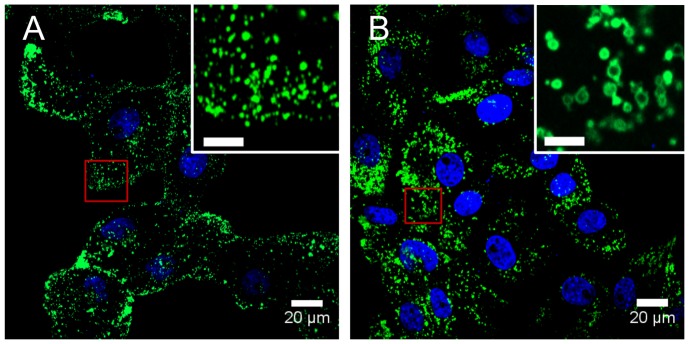
Incubation of cells with sucrose results in enlargement of lysosomes. (*A*) Confocal fluorescence microscopy image of untreated BS-C-1 cells shows the normal cellular distribution and punctate appearance of lysosomes (green) labeled with EYFP. The nuclei are stained with DAPI (blue). (*B*) Incubation with sucrose (50 mM, 12 h) leads to enlargement of lysosomes. The increased diameter gives the lysosomes a circular appearance. The inset shows an expanded view of the region in the red box. The scale bar in the inset is 5 *µ*m.

Lysosomes in monkey kidney epithelial cells (BS-C-1) were fluorescently labeled with enhanced yellow fluorescent protein (EYFP) fused to the cytosolic component of lysosome associated membrane protein 1 (LAMP1-EYFP, Figure 1A). The BS-C-1 cells used in these experiments were engineered previously to stably express LAMP1-EYFP [21], [22].

Lysosomes were enlarged by incubating cells with sucrose (12 h, 50 mM), which leads to an osmotic swelling of the lysosomes (Figure 1B) [16]–[18]. The increased diameter of the lysosomes is observed in fluorescence microscopy images as a population of circular, rather than punctate, lysosomes. In addition to the increased diameter, a subset of the lysosomes form clusters after incubation with sucrose. Clustered lysosomes were not analyzed as the clusters themselves can inhibit motion.

Experiments were also carried out in human cervix epithelial cells (HeLa) to ensure that results were not cell type dependent. A similar sucrose-dependent swelling of lysosomes was observed in HeLa cells (Figure S1), although a longer incubation period was required (24 h, 50 mM).

### Measurement of Lysosome Diameter

**Figure 2:**
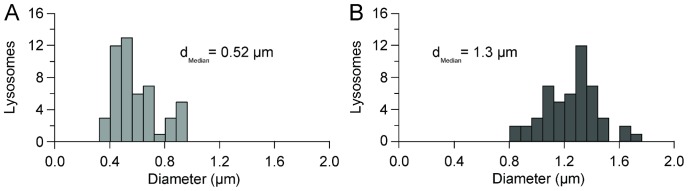
Distribution of lysosome diameters. (*A*) Distribution of lysosome diameters measured in control, untreated cells. (*B*) Incubation with sucrose shifts the distribution of lysosome diameters to greater values. For both plots, n = 50 lysosomes from 3 cells.

Lysosome diameter was measured using ImageJ to analyze confocal fluorescence microscopy images (Figure 2). Untreated cells have punctate lysosomes with a median diameter of 520 nm measured for 50 lysosomes in 3 cells (Figure 2A). The caveat associated with this measurement is that the diameter of the punctate lysosomes approaches the point spread function of the confocal microscope used for these experiments (300 nm). Circular lysosomes were not observed in untreated cells. In sucrose-treated cells, the circular lysosomes have a median diameter of 1.3 *µ*m (n = 50 lysosomes, 3 cells), with 67% of lysosomes, averaged over 3 cells, having a circular appearance (Figure 2B).

We also tested whether longer incubation times and higher concentrations of sucrose (100 mM, 24 hrs) would lead to even larger lysosomes (data not shown). We found that a greater percent of lysosomes were enlarged, but the median diameter remained the same.

### Lysosomal Motion Includes Active and Passive Transport

**Figure 3:**
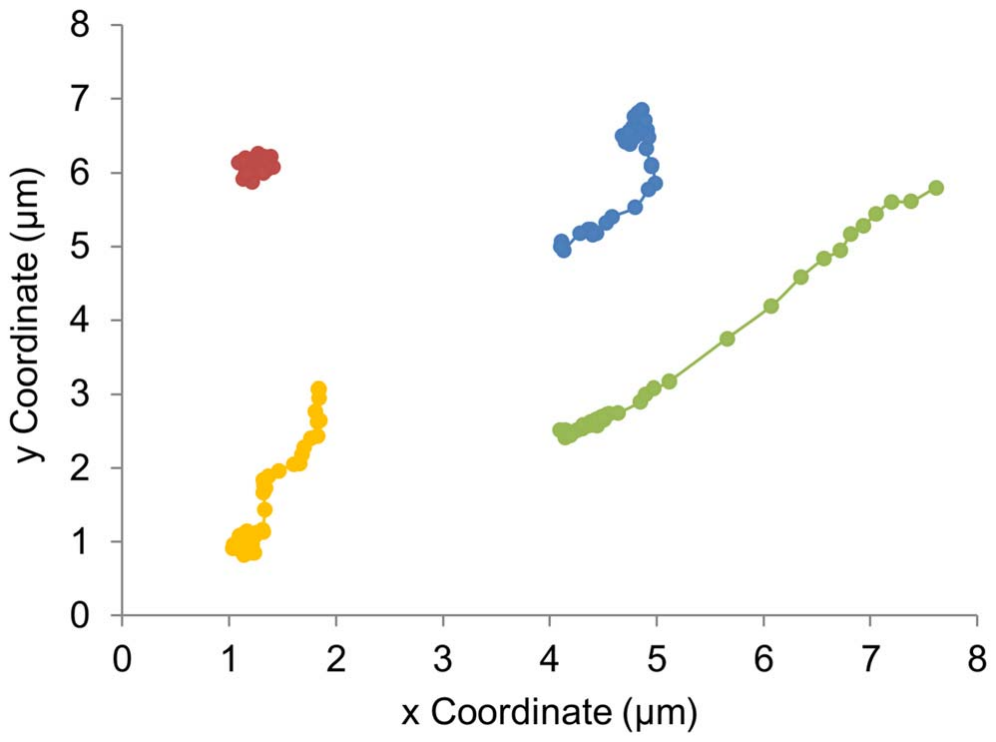
Lysosomes undergo both active transport and diffusive motion. Representative trajectories of 4 untreated lysosomes. Trajectories show periods of long-range transport and periods of diffusion. An image was recorded every 0.3 seconds.

Lysosomal motion consists of active, ATP-dependent, transport along microtubules and periods of diffusion [3]–[6]. This is observed in individual trajectories as stretches of long-range, directed motion interspersed with random, diffusive motion (Figure 3). The enlarged lysosomes, with a ∼2x increase in diameter, provide an ideal system to determine how increased lysosome diameter affects intracellular active transport.

### Increased Lysosome Diameter Decreases Lysosome Diffusion

**Figure 4:**
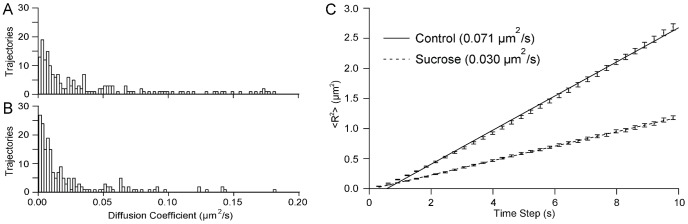
Increased lysosome diameter decreases diffusive lysosome motion. (*A*) Diffusion coefficients from 200 punctate lysosomes in 7 untreated cells. (*B*) Diffusion coefficients from 200 enlarged lysosomes in 7 sucrose-treated cells. (*C*) Averaged MSDs from the lysosomes shown in (*A*) and (*B*). Both MSD curves are fit to a line with a slope of 4D. Error bars show standard error.

LAMP1-EYFP was used as a fluorescent tag to track the motion of lysosomes. Cells were maintained at 37°C during imaging. Images were recorded every 0.3 s and MATLAB scripts were used to analyze the position of individual lysosomes as a function of time. Free diffusion is characterized by a linear mean square displacement (MSD) with a slope of 4D. An analysis of 200 lysosomes from 7 untreated cells shows that normal lysosomes diffuse with a broad range of diffusion coefficients (Figure 4A). Averaging the MSDs of the 200 lysosomes results in an average diffusion coefficient of 0.071 *µ*m^2^ s^−1^ for lysosomes in untreated cells (Figure 4C). In comparison, the diffusion coefficients of the enlarged lysosomes in sucrose-treated cells show a narrowed distribution shifted to lower values (Figure 4B). The diffusion coefficient calculated from averaged MSDs decreases to 0.030 *µ*m^2^ s^−1^ (n = 200 lysosomes, 7 cells, Figure 4C). As sucrose-treatment does not enlarge all lysosomes, it was also possible to compare punctate and enlarged lysosomes, both within sucrose-treated cells, which showed a similar trend (Figure S2). Similar results were obtained for lysosomes in HeLa cells (Figure S3).

### Active Transport is not Affected by Lysosome Diameter

Active transport was defined as periods of directed lysosome motion in a single direction rather than the random motion associated with diffusion. Periods during which lysosomes moved in a single direction for a minimum of 1.5 s were considered active. The velocity at each step was recorded. It is important to note that this measure of velocity is not that of a single motor protein step, but rather the distance traveled by the lysosome between frames. Lysosomes moving towards the cell periphery were classified as undergoing anterograde motion. Those moving towards the nucleus were classified as retrograde with velocity multiplied by ^−^1 to denote the direction. For both control and sucrose-treated cells, 20 lysosomes in 5 cells were analyzed.

**Figure 5:**
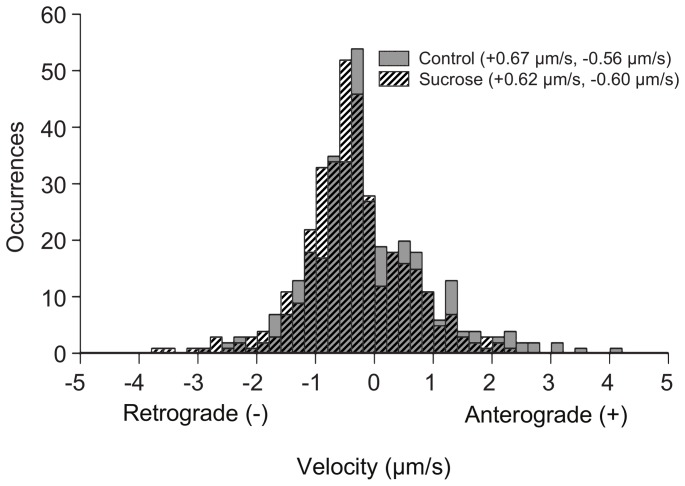
Active transport is unaffected by increased lysosome diameter. Histogram showing the distribution of lysosome velocities for punctate lysosomes in control cells (solid gray) and enlarged lysosomes in sucrose-treated cells (black stripes) cells. Data for each distribution was obtained from 20 lysosomes in 5 cells.

Both control and sucrose-treated cells show a wide distribution of lysosome velocities (Figure 5). In untreated cells, lysosomes moving towards the cell periphery (anterograde) had a median velocity of +0.67 *µ*m s^−1^±0.79 *µ*m s^−1^. The enlarged lysosomes show a similarly broad distribution with a median velocity of +0.62 *µ*m s^−1^±0.52 *µ*m s^−1^. Transport toward the cell nucleus (retrograde) had a median velocity of −0.56 *µ*m s^−1^± −0.53 *µ*m s^−1^ for untreated lysosomes and −0.60 *µ*m s^−1^± −0.61 *µ*m s^−1^ for enlarged lysosomes in sucrose-treated cells.

## Discussion

The goal of this research was to determine how lysosome diameter affects lysosome transport. Early research in the field of intracellular transport examined transport of small (100 nm - 200 nm), medium (200 nm - 600 nm), and large (800 nm –5 µm) organelles in squid axons and found slower mean velocities for the larger organelles [23]. However, these were not all the same organelles. The largest organelles were mitochondria, the smaller organelles were not identified. Incubating cells with sucrose results in enlarged lysosomes (Figure 1), allowing us to compare transport of identical organelles with different diameters within live cells.

The increased diameter of the lysosomes, likely an osmotic effect [16]–[18], gives them a circular appearance resulting from the LAMP1-EYFP fluorescent tag on the membrane of the lysosome. We use this difference in morphology to group lysosomes into two categories: punctate (small) and circular (large). While there are certainly larger punctate lysosomes and smaller circular lysosomes, this binning provides a method to separate lysosomes based on size during single particle tracking. Previous measurements of lysosome diameter range from 50 nm to 500 nm [1]. For example, mouse macrophage lysosomes have an average diameter of 136 nm [24]. Rat kidney fibroblasts have lysosomes with diameters of 300 nm to 400 nm [25]. Untreated, punctate, lysosomes in these monkey kidney epithelial cells have a median diameter of 520 nm (Figure 2A). This is likely an over-estimate as smaller lysosomes would be below the 300 nm point spread function of the confocal microscope used to obtain images. Enlarged, circular, lysosomes have median diameters of 1.3 µm following sucrose treatment (Figure 2B). The approximately 2-fold increase in diameter of lysosomes following sucrose incubation provides an ideal system for the comparison of identical organelles with different diameters.

The motion of lysosomes includes both active transport and passive diffusion (Figure 3). The relationship between lysosome diameter and diffusive and active transport was examined by comparing the motion of punctate and enlarged lysosomes (Figures 4 and 5). The enlarged lysosomes showed a 2.4x decrease in intracellular diffusion coefficient, in good agreement with the 2.5x increase in diameter. The average diffusion coefficients can be used to calculate the average viscosity of the cytosol using the Stokes-Einstein relation; D = k_b_T/6πηr with r as the median radius of the lysosomes measured from confocal microscopy, 260 nm and 650 nm for untreated punctate lysosomes and enlarged, sucrose-treated lysosomes, respectively. The diffusion of lysosomes in the untreated cells reflects a viscosity of 12 cP; sucrose-treated cells have viscosity of 9 cP. Previous measurements of cytosol viscosity range from being equal to that of water (1 cP) to 2000x greater than water [26]–[31]. Additionally, it is known that cytosol viscosity varies as function of sub-cellular environment and cell cycle [7], [8]. Our measurement of 9–12 cP is similar to that obtained for gold nanoparticles (<5 nm diameter) and quantum dots (26 nm diameter), which reported cytosolic viscosities of 20 cP (average) and 4–200 cP, respectively [30], [31]. Most similar to our system are measurements of the intracellular motion of endosomes containing cholera toxin, which showed a viscosity of 5 cP based on the diffusion coefficient, measured with fluorescence correlation spectroscopy, and an estimate of endosome diameter [32]. Microrheology experiments tracking 100 nm polystyrene nanoparticles show much larger cytosolic viscosities; 800–1800 cP in fibroblasts cells [28], [29]. These microrheology experiments are, in some sense, the gold standard for measuring cytosol viscosity. It is interesting to note that viscosities measured from the microrheology experiments are ∼100x greater than the viscosities obtained for lysosomes or endosomes. While averaging over a greater number of lysosomes on wider timescales could shift our values, it is also possible that organelles, such as lysosomes, interact differently with the cytosol than do polystyrene nanoparticles, resulting in a lower effective viscosity.

Lysosomes undergoing active transport moved with a broad distribution of velocities (Figure 5). The small, punctate, lysosomes in untreated cells had a median value of +0.67* µ*m s^−1^ during anterograde transport and −0.56 *µ*m s^−1^ during retrograde transport. Large, circular, lysosomes in sucrose-treated cells had median velocities of +0.62* µ*m s^−1^ and −0.60* µ*m s^−1^, respectively. The velocities measured in the current experiments are similar to lysosome speeds of 0.2 *µ*m s^−1^ measured previously within our lab [33], as well speeds of 0.45 *µ*m s^−1^ and 0.41 *µ*m s^−1^ for retrograde and anterograde transport, respectively, measured in recent single particle tracking experiments carried out in the same BS-C-1 cells [6]. This range of velocities is also similar to those obtained for endosomes in CHO cells, which had maximum velocities of 4 *µ*m s^−1^ [34]. Overall, lysosomes move with a broad range of velocities independent of lysosome diameter. Based on a KS test to compare distributions, our results do not show any significant difference in active transport as a function of diameter. This suggests that cytosolic drag is not a major factor in active transport.

In summary, these experiments are the first to measure the influence of lysosome diameter on lysosome transport within living cells. Increasing the diameter of lysosomes provides a method to address fundamental questions of intracellular transport. As expected, we find that enlarged lysosomes have decreased diffusion. Active transport of lysosomes by motor proteins along microtubules is not affected by lysosome diameter. These results have important implications in fundamental questions of intracellular transport and may provide insight into lysosome-based diseases.

## Materials and Methods

### Cell Culture

BS-C-1 monkey kidney epithelial cells (CCL-26, ATCC, Manassas, VA) transfected to stably express LAMP1-EYFP have been described previously [21], [22]. The cells were maintained in a 37°C, 5% carbon dioxide environment in Minimum Essential Medium (MEM, 61100061, Invitrogen, Grand Island, NY) with 10% (v/v) fetal bovine serum (FBS, 10437028, Invitrogen) supplemented with 200 *µ*g/mL G418 (345810, Calbiochem, Dornstadt, Germany) and passaged every 4 days. For fluorescence microscopy, cells were cultured in 35 mm glass-bottom cell culture dishes (P35G-1.5-14-C, MatTek, Ashland, MA). For live cell imaging, the growth medium was replaced with Leibovitz’s L-15 medium (21083-027, Invitrogen). Nuclei were stained with 27 *µ*M 4′,6-diamidino-2-phenylindole dilactate (DAPI, D3571, Invitrogen) at 37°C in growth medium for 30 minutes. For static imaging, cells were fixed with 4% (v/v) formaldehyde (28908, Thermo Scientific, Rockford, IL) and imaged in PBS. For sucrose-mediated enlargement of lysosomes, BS-C-1 cells were incubated with 50 mM sucrose (4072–01, J. T. Baker, Phillipsburg, NJ) in MEM containing 10% (v/v) FBS for 12 h. A description of the HeLa cell culture is provided in Text S1.

### Confocal Microscopy

Confocal microscopy images were collected with a FluoView 1000 laser scanning confocal microscope (Olympus, Hunt Optics and Imaging, Pittsburgh, PA) using a 1.42 NA, 60x, oil immersion objective. EYFP was excited with the 514 nm line of an argon ion laser. DAPI was excited with a 405 nm solid state laser. The 535–565 nm and 430–470 nm band pass filters were used to filter emission for EYFP and DAPI, respectively. For all images, the pinhole was set to obtain a 1 *µ*m thick optical slice. Static images were acquired using Kalman integration with an integration count of 10 for each image. For live cell imaging, the cells were maintained at 37°C using a stage top incubator and objective heater (Tokai Hit, Fujinomiya-shi, Shizuoka-ken, Japan). Images were collected in one way scanning mode with a sampling speed of 2 µs/pixel and a frame captured every 0.307 s.

### Data Analysis

Lysosome diameter was measured using ImageJ (http://rsb.info.nih.gov/ij/) with lysosomes approximated as circles. The selection of circular, enlarged, lysosomes was carried out using a size mask of 0.5 *µ*m^2^ to 5.0 *µ*m^2^. Punctate lysosomes were selected with a size mask of 0.1 *µ*m^2^ to 1.5 *µ*m^2^. Thresholds were kept the same for all images. Clustered lysosomes were not included in the size analysis. Particle detection and trajectory linking was carried out using the u-track MATLAB (The MathWorks, Natick, MA) software package [35].

For diffusive motion, the average mean square displacement (MSD) curve of 200 randomly selected lysosomes was fit to a line with a slope of 4D where D is the diffusion coefficient. Standard error was calculated and then propagated through to the average MSD curves. Trajectory segments showing consistent motion toward or away from the nucleus for more than 5 frames were considered active transport. Velocities were measured as the frame to frame displacement per second. Velocity distribution were compared using a Kolmogorov-Smirnov test (KS test). A p-value of 0.01 was used as the threshold to determine if two data sets had the same distribution. The null hypothesis is that the two data sets for comparison have the same distribution. A p-value below 0.01 rejects the null hypothesis indicating that the two data sets are from different distributions.

## Supporting Information

Figure S1 **Sucrose-mediated enlargement of lysosomes in HeLa cells.** (*A*) Confocal fluorescence microscopy image of untreated HeLa cells shows the normal cellular distribution and punctate appearance of lysosomes (green) labeled with EYFP. The nuclei are stained with DAPI (blue). (*B*) Incubation with sucrose (50 mM, 24 h) leads to enlargement and clustering of lysosomes. The increased diameter gives the lysosomes a circular appearance. The inset shows an expanded view of the region in the red box. The scale bar in the inset is 2 µm.
(TIF)

Figure S2 **Diffusion coefficients of punctate and enlarged lysosomes in sucrose-treated cells.** Averaged MSDs from 50 punctate and 50 enlarged lysosomes in 10 sucrose-treated cells. Both MSD curves are fit to a line with a slope of 4D. Error bars show standard error. The ∼2x decrease in diffusion coefficient for enlarged lysosomes is identical to that observed for enlarged lysosomes in sucrose-treated cells compared to punctate lysosomes in untreated cells (Figure 4). The absolute values are a function of cell passage number and cell confluency. The viscosity of 50 mM sucrose is nearly identical to that of water (CRC Handbook of Chemistry and Physics 91^st^ Edition, 2010) making it unlikely that the decreased diffusion coefficient of the enlarged lysosomes in sucrose-treated cells (Figure 4) is an artifact of increased cytosolic viscosity. These results confirm that sucrose treatment does not affect the overall viscosity of the cell.
(TIF)

Figure S3 **Increased lysosome diameter decreases diffusive lysosome motion in HeLa cells.** (*A*) Diffusion coefficients from 200 punctate lysosomes in 3 untreated cells. (*B*) Diffusion coefficients from 200 enlarged lysosomes in 4 sucrose-treated cells. (*C*) Averaged MSDs from the lysosomes shown in (*A)* and (*B)*. Both MSD curves are fit to a line with a slope of 4D. Error bars show standard error.
(TIF)

Text S1 **Supporting Information.**
(DOCX)
