# Supplementary figures and images for: Lysosome Transport as a Function of Lysosome Diameter
Source: PLoS One. 2014 Jan 31;9(1):e86847. doi: 10.1371/journal.pone.0086847 (PMC3908945; doi:10.1371/journal.pone.0086847)

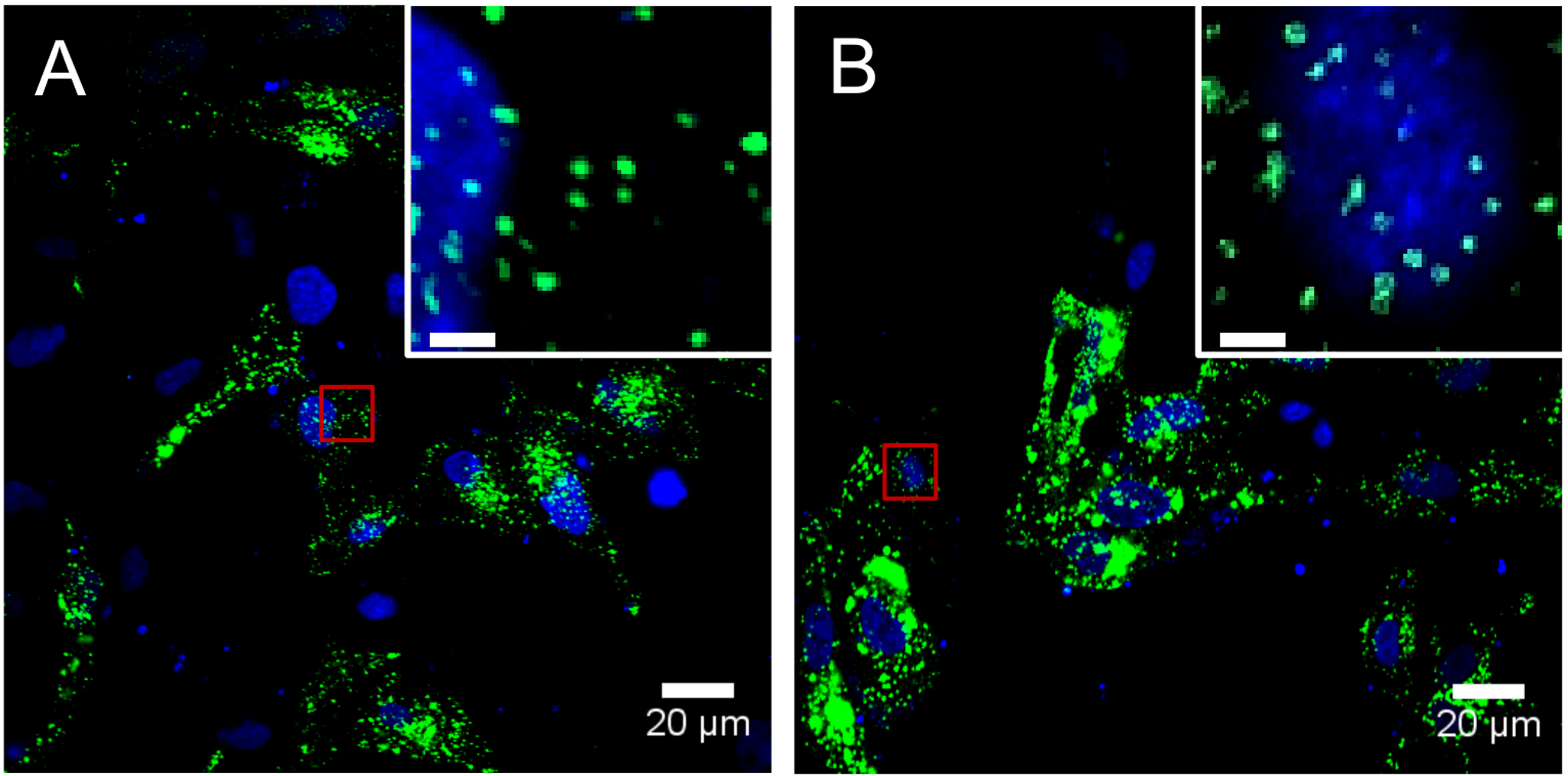

Supplement: Figure S1 — Sucrose-mediated enlargement of lysosomes in HeLa cells. (A) Confocal fluorescence microscopy image of untreated HeLa cells shows the normal cellular distribution and punctate appearance of lysosomes (green) labeled with EYFP. The nuclei are stained with DAPI (blue). (B) Incubation with sucrose (50 mM, 24 h) leads to enlargement and clustering of lysosomes. The increased diameter gives the lysosomes a circular appearance. The inset shows an expanded view of the region in the red box. The scale bar in the inset is 2 µm. (TIF) [file pone.0086847.s001.tif]

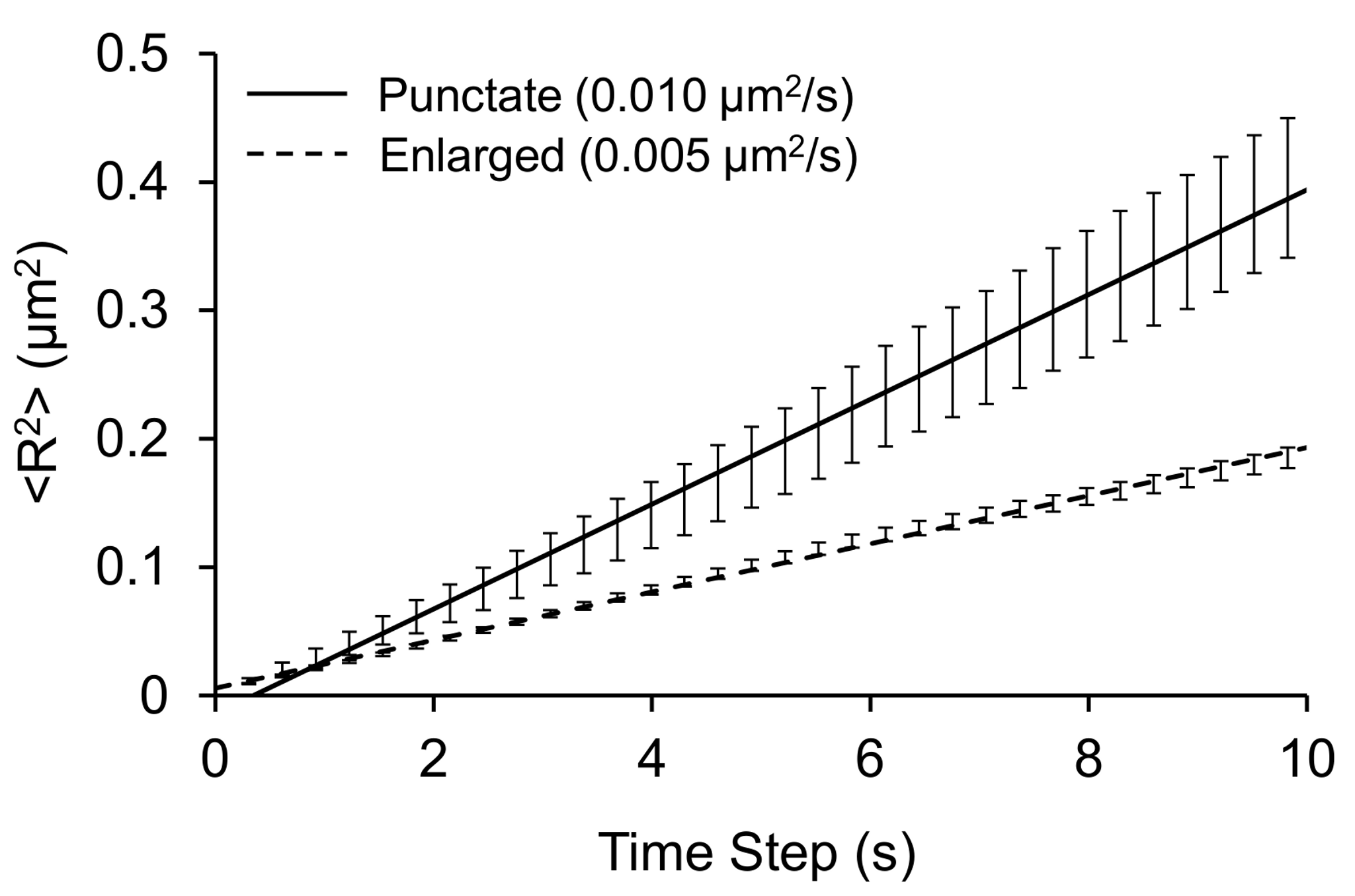

Supplement: Figure S2 — Diffusion coefficients of punctate and enlarged lysosomes in sucrose-treated cells. Averaged MSDs from 50 punctate and 50 enlarged lysosomes in 10 sucrose-treated cells. Both MSD curves are fit to a line with a slope of 4D. Error bars show standard error. The ∼2x decrease in diffusion coefficient for enlarged lysosomes is identical to that observed for enlarged lysosomes in sucrose-treated cells compared to punctate lysosomes in untreated cells (Figure 4). The absolute values are a function of cell passage number and cell confluency. The viscosity of 50 mM sucrose is nearly identical to that of water (CRC Handbook of Chemistry and Physics 91st Edition, 2010) making it unlikely that the decreased diffusion coefficient of the enlarged lysosomes in sucrose-treated cells (Figure 4) is an artifact of increased cytosolic viscosity. These results confirm that sucrose treatment does not affect the overall viscosity of the cell. (TIF) [file pone.0086847.s002.tif]

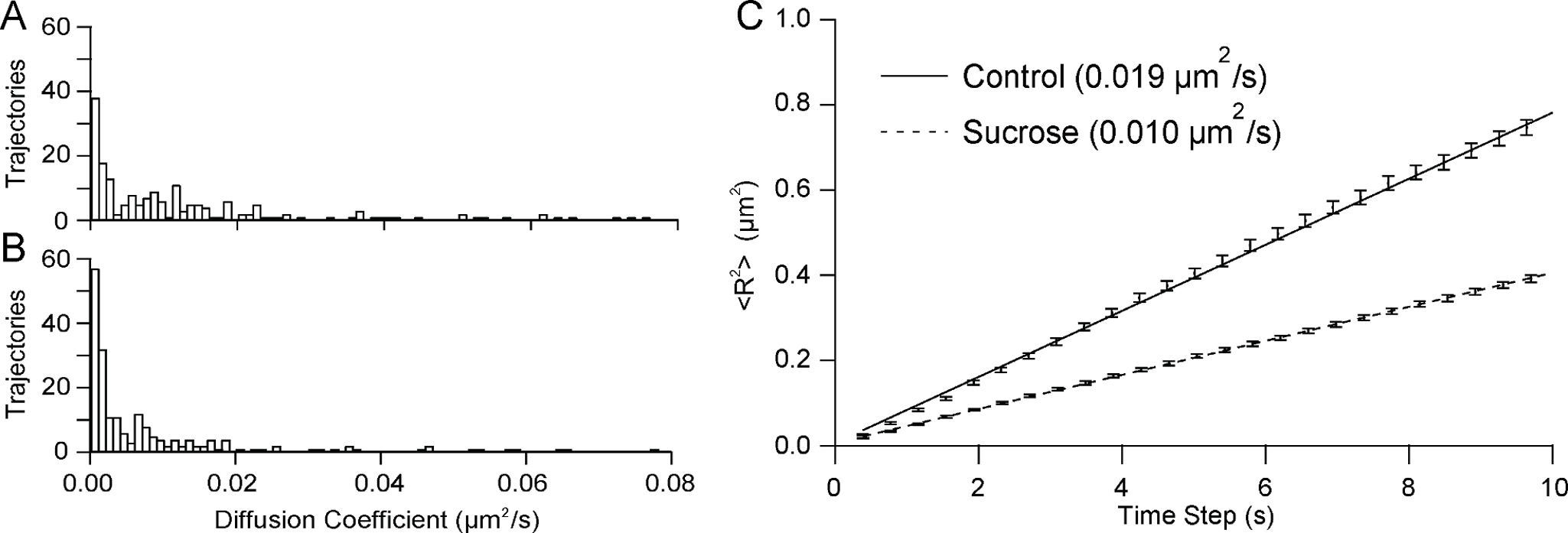

Supplement: Figure S3 — Increased lysosome diameter decreases diffusive lysosome motion in HeLa cells. (A) Diffusion coefficients from 200 punctate lysosomes in 3 untreated cells. (B) Diffusion coefficients from 200 enlarged lysosomes in 4 sucrose-treated cells. (C) Averaged MSDs from the lysosomes shown in (A) and (B). Both MSD curves are fit to a line with a slope of 4D. Error bars show standard error. (TIF) [file pone.0086847.s003.tif]
