# Supplementary material for: Lysosome Transport as a Function of Lysosome Diameter
Source: PLoS One. 2014 Jan 31;9(1):e86847. doi: 10.1371/journal.pone.0086847 (PMC3908945; doi:10.1371/journal.pone.0086847)
Supplement: Text S1 — Supporting Information. (DOCX) [file pone.0086847.s004.docx]

**Supporting Information**

**Lysosome Transport as a Function of Lysosome Diameter**

Debjyoti Bandyopadhyay^1^, Austin Cyphersmith^1^, Jairo A. Zapata^1^, Y. Joseph Kim^1^*,* and Christine K. Payne^1,^*

^1^School of Chemistry and Biochemistry and Petit Institute for Bioengineering and Bioscience,

Georgia Institute of Technology, Atlanta, Georgia, USA

Key words: lysosome; endocytosis; motor proteins; cytosolic drag; fluorescence microscopy; single particle tracking

* Corresponding author: christine.payne@chemistry.gatech.edu

***Cell Culture***

HeLa cells (ATCC, Manassas, VA) were maintained in a 37 °C, 5% carbon dioxide environment in Minimum Essential Medium (MEM, 61100061, Invitrogen, Grand Island, NY) with 10% (v/v) fetal bovine serum (FBS, 10437028, Invitrogen) and passaged every 3 days. For fluorescence microscopy, cells were cultured in 35 mm glass-bottom cell culture dishes (P35G-1.5-14-C, MatTek, Ashland, MA). For live cell imaging, the growth medium was replaced with Leibovitz’s L-15 medium (21083-027, Invitrogen). Nuclei were stained with 27 *μ*M 4’,6-diamidino-2-phenylindole dilactate (DAPI, D3571, Invitrogen) at 37 °C in complete growth medium for 30 minutes. For the measurement of lysosome diameter, cells were fixed with 4% (v/v) formaldehyde (28908, Thermo Scientific, Rockford, IL) and imaged in PBS.

***Transient expression of LAMP1-EYFP in HeLa cells***

Lysosomes in HeLa cells were fluorescently labeled by transiently expressing LAMP1-EYFP. Transfection was carried out by adding 92 µL of MEM (51200-038, Invitrogen, Grand Island, NY) containing 6 µL FuGENE 6 transfection reagent (E2693, Promega, Madison, WI) and 2 µg of plasmid DNA (1816, Addgene, Cambridge, MA) to a 60-70% confluent monolayer of cells grown on optical dishes in 1 mL of complete growth medium (MEM containing 10% FBS). After 24 h, the transfection medium was replaced with normal full growth medium and the cells were imaged after an additional 24 h.

***Confocal microscopy***

Confocal microscopy was carried out as described in the main text with one change: live cell imaging used sampling speeds of 2.0 µs/pixel and 4.0 µs/pixel with a frame captured every 0.388 s and 0.385 s, respectively.
